# Eosinophilic esophagitis after induction of oral tolerance for cow’s milk proteins

**DOI:** 10.1186/2045-7022-3-S3-P9

**Published:** 2013-07-25

**Authors:** C Muñoz Archidona, JF Viada Bris, SJ Quevedo Teruel, S Fernández Fernández, AI Rayo Fernández, T Bracamonte Bermejo, LA Echeverría Zudaire

**Affiliations:** 1 Pediatrics, Hospital Severo Ochoa, Leganés, Madrid, Spain

## Background

The incidence of confirmed cow´s milk protein (CMP) allergy in infancy is about 2-5% in developed countries. Until now, management strategies included only avoiding CMP intake, because no treatment was available. However, new therapeutic strategies have emerged in the last years. The most important is the induction of oral tolerance (IOT), with results which are promising, improving the quality of life, above all in those who can suffer a severe allergic reaction. Nevertheless, more studies about long-term effects are needed. We describe 4 cases of eosinophilic esophagitis (EoE) after IOT.

## Methods

We evaluated 59 patients with IgE-mediated allergy for CMP on whom we administered IOT treatment according to our protocol. Once a dose of 200ml/day was achieved, a continued daily dose was taken at home. We did a clinical follow-up at 6 months and then annually, with skin prick tests and specific IgE levels. We asked specifically about digestive symptoms.

## Results

43 patients were followed for at least one year. 4 presented digestive symptoms and were subsequently diagnosed of EoE. Case 1: Received IOT for 149 days. After 47 months taking a daily milk dose, he began with abdominal pain and vomiting. Endoscopy and biopsies performed were compatible with EoE. Case 2: Received IOT for 130 days. He was in medical follow-up on suspicion of celiac disease. He had no digestive symptoms. An endoscopy performed 13 months later, confirmed EoE and celiac disease. He had a normal endoscopy performed 2 years before IOT. Case 3: Received IOT for 112 days. After 3 months, he began with vomiting, epigastralgia and pyrosis, which improved reducing the dose of cow's milk. 12 months later, biopsies taken endoscopically confirmed EoE. Case 4: Received IOT for 112 days. 17 months later, he developed symptoms of gastroesophageal reflux and the endoscopy showed esophageal, gastric and duodenal mucosa affection, with an increase in the number of eosinophils in these locations.

## Conclusion

Although it is difficult to prove a relationship between IOT and EoE, we should inquire about the appearance of digestive symptoms in those patients that completed an IOT. We must emphasize the fact that none of the patients with EoE wanted to suspend the daily milk intake due to the improvement experienced in their quality of life after IOT. It is very important to follow-up all patients to detect possible long-term effects.

## Disclosure of interest

None declared.

